# Formation and Transfer of Multi-Species Biofilms Containing *E. coli* O103:H2 on Food Contact Surfaces to Beef

**DOI:** 10.3389/fmicb.2022.863778

**Published:** 2022-05-30

**Authors:** Yuchen Nan, Argenis Rodas-Gonzalez, Kim Stanford, Celine Nadon, Xianqin Yang, Tim McAllister, Claudia Narváez-Bravo

**Affiliations:** ^1^Department of Food and Human Nutritional Sciences, University of Manitoba, Winnipeg, MB, Canada; ^2^Department of Animal Science, University of Manitoba, Winnipeg, MB, Canada; ^3^Department of Biological Sciences, University of Lethbridge, Lethbridge, AB, Canada; ^4^National Microbiology Laboratory, Public Health Agency of Canada, Winnipeg, MB, Canada; ^5^Agriculture and Agri-Food Canada, Lacombe Research and Development Centre, Lacombe, AB, Canada; ^6^Agriculture and Agri-Food Canada, Lethbridge Research and Development Centre, Lethbridge, AB, Canada

**Keywords:** STEC, multispecies biofilm, beef contamination, persistence, dry biofilm

## Abstract

Interactions of Shiga toxin–producing *E. coli* (STEC; O103:H2) with lactic acid bacteria (LAB) or spoilage bacteria (SP) multispecies biofilms on polyurethane (TPU) and stainless-steel (SS) were assessed at 10 and 25°C under wet and dry conditions after 6, 30, and 60 days of storage. One LAB T1: *Carnobacterium piscicola* + *Lactobacillus bulgaricus*, and two SP T2: *Comamonas koreensis* + *Raoultella terrigena;* T3: *Pseudomonas aeruginosa* + *C. koreensis* were assessed for their ability to form multispecies biofilms with O103:H2. O103:H2 single-species biofilms served as a control positive (T4). Coupons were stored dry (20–50% relative humidity; RH) or moist (60–90% RH) for up to 60 days, at which point O103:H2 transfer to beef and survival was evaluated. At 25°C, T3 decreased beef contamination with O103:H2 by 2.54 log_10_ CFU/g (*P* < 0.001). Overall, at 25°C contamination of beef with O103:H2 decreased (*P* < 0.001) from 3.17 log_10_ CFU/g on Day 6 to 0.62 log_10_ CFU/g on Day 60. With 60 days dry biofilms on TPU, an antagonistic interaction was observed among O103:H2 and multispecies biofilm T1 and T3. *E. coli* O103:H2 was not recovered from T1 and T3 after 60 days but it was recovered (33%) from T2 and T4 dry biofilms. At 10°C, contamination of beef with O103:H2 decreased (*P* < 0.001) from 1.38 log_10_ CFU/g after 6 days to 0.47 log_10_ CFU/g after 60 days. At 10°C, recovery of O103:H2 from 60 days dry biofilms could only be detected after enrichment and was always higher for T2 than T4 biofilms. Regardless of temperature, the transfer of O103:H2 to beef from the biofilm on TPU was greater (*P* < 0.001) than SS. Moist biofilms also resulted in greater (*P* < 0.001) cell transfer to beef than dry biofilms at 10 and 25°C. Development of SP or LAB multispecies biofilms with O103:H2 can either increase or diminish the likelihood of beef contamination. Environmental conditions such as humidity, contact surface type, as well as biofilm aging all can influence the risk of beef being contaminated by STEC within multi-species biofilms attached to food contact surfaces.

## Introduction

Shiga toxin–producing *Escherichia coli* (STEC) are important enteric pathogens linked to outbreaks involving meat and produce and are a worldwide health concern (Nguyen and Sperandio, 2012; CDC, 2014; U.S. Department of Health and Human Services, 2014). The prevalence of Shiga Toxigenic *E. coli* (STEC) in Canadian cattle was evaluated at two western Canadian slaughter plants. In this research, fecal samples (*n* = 1,794) were collected for 2 years from cattle trailers. Results showed that 94.4% of the fecal samples were positive for the serogroup O103 followed by O45 (93.1%), O26 (82.3%), O157 (78.8%), O121 (66.1%), O111 (8.2%), and O145 (7.0%) (Stanford et al., 2016). Ruminants are the main reservoir of STEC which can be transferred from hides and feces to the carcasses during processing (Bryan et al., 2015; PHAC, 2015). As few as 10 STEC cells can cause illness in humans which may develop into serious complications such as hemorrhagic colitis and hemolytic uremic syndrome (Etcheverria et al., 2010; PHAC, 2015). In 2019, 1,462 STEC infections were reported to the Canadian National Enteric Surveillance Program (NESP) with approximately 73% of these caused by non-O157 (PHAC, 2020). Non-O157 serogroups causing disease in Canada have exceeded the number of O157-related since 2017 (PHAC, 2020). In 2019, non-O157 isolated from human infections were primarily represented by five serogroups: O26 (16%), O111 (10%), O103 (8%), O118 (3%), and O121 (3%) (PHAC, 2020).

Biofilm is a community of microorganisms attached to a solid surface or each other (Srey et al., 2013; Vogeleer et al., 2014; Adator et al., 2018). Bacterial cells within biofilms are embedded within a self-produced extracellular polymeric matrix (EPM), which reduces their sensitivity to selective pressures such as heat, biocides, and antimicrobials (Srey et al., 2013; Vogeleer et al., 2014; Adator et al., 2018). Consequently, biofilms contribute to the persistence of *E. coli* on beef fabrication equipment (Yang et al., 2018). Biofilms are prominent within beef processing facilities, as more than 80% of *E. coli* isolated from beef fabrication equipment formed strong biofilms on stainless steel and were highly resistant to quaternary ammonium chloride (Yang et al., 2018). Of the top seven STEC isolates (*n* = 745), 93% of those collected from live cattle lacked biofilm-forming ability (Stanford et al., 2021). Therefore, STEC is likely to establish its presence on beef fabrication equipment through biofilm formation, even though biofilm-forming STEC are rare (Stanford et al., 2021). For example, research looking at STEC collected from live cattle showed that 93% (*n* = 745) lacked biofilm-forming ability (Stanford et al., 2021). Despite only 3% weak, 3% intermediate, 1% strong, and 0.3% extreme biofilm-forming STEC among the 745 isolates from cattle, they may be related to HEP through surviving the sanitation process and might be persistent in the beef fabrication facility (Stanford et al., 2021). Interestingly, multiple previous studies demonstrated that non-pathogenic bacteria originating from beef facilities such as *Comamonas testosterone* (Marouani-Gadri et al., 2009) and *Acinetobacter calcoaceticus* (Habimana et al., 2010) can enhance STEC O157:H7 biofilm formation.

Shiga toxin–producing *E. coli* biofilm on contact surfaces has generally been investigated using single-species biofilms in wet conditions (Wang et al., 2012; Adator et al., 2018; Yang et al., 2018). However, biofilms that form within beef processing facilities are typically composed of multiple species (Wang et al., 2018; Visvalingam et al., 2019a,b) and these biofilms can exist in wet or dry conditions. For example, both lactic acid bacteria (LAB) (e.g., *Carnobacterium* spp.) and spoilage bacteria (e.g., *Raoultella* spp., *Pseudomonas* spp.) were isolated from conveyor belt biofilms within a beef-processing facility (Wang et al., 2018). Functional characteristics of bacteria within multispecies biofilms can substantially differ from that exhibited within single-species biofilms (Burmolle et al., 2014; Pang and Yuk, 2018). Thus, to enhance risk assessment tools and improved pathogen intervention strategies, it is important to investigate the interactions of STEC with other bacterial species within biofilms (Giaouris et al., 2015) and the variables that might be impacting biofilm formation. The objectives of this study were (1) to evaluate potential synergistic and antagonistic interactions of STEC (O103:H2) with either LAB or spoilage bacteria (SP) within multispecies biofilms formed on thermoplastic polyurethane (TPU) or stainless steel (SS); (2) to determine the extent of transfer of O103:H2 cells from single and multispecies biofilms to beef with different storage times, temperatures, and humidity; and (3) to determine the capacity of STEC to survive within single vs. multispecies biofilms.

## Materials and Methods

### Bacteria Strains and Culture Conditions

A total of 9 STEC strains including 7 serogroups, 12 SP, and 12 LAB were assessed for their suitability to use in this study (Table 1). STEC strains were cultured on MacConkey agar plates (Hardy Diagnostics Inc., Santa Maria, CA, United States), while SP and LAB bacteria were cultured on Trypticase Soy Agar (TSA; Becton, Dickinson and Company, MD, United States) at 25°C. A single colony of each STEC, LAB, and SP strain was transferred from each plate into individual 10 ml Lennox broth with no salt (LB-NS; Tryptone 10 g/L and yeast extract 5 g/L) and grown to a cell density of 10^8^ CFU/ml. The incubation time required for the strains to reach early stationary phase varied from 24 to 72 h (Visvalingam et al., 2019a). Cultures were subsequently diluted to 10^6^ CFU/ml for use in biofilm formation assessment assays.

**TABLE 1:** STEC, LAB, and spoilage bacteria which selected to perform the biofilm-forming ability test.

Serotype	Strain ID	Source	Category
O26: H11	00-3941	Human	STEC
O45: H7	05-6545	Human	STEC
O103: H2	99-2076	Human	STEC
O111: NM	CFS3	Human	STEC
O121: H19	03-2832	Human	STEC
O145: H2	75-83	Human	STEC
O157: H7	1934	Beef	STEC
O157: H7	1931	Hamburger	STEC
O157: H7	R508	Bovine/feces	STEC
*Lactobacillus sakei*	S19	Vacuum-packaged meat	LAB
*Leuconostoc gelidum*	S21	Vacuum-packaged meat	LAB
*Carnobacterium divergens*	B1	Vacuum-packaged meat	LAB
*Carnobacterium maltaromaticum*	LAB9_67	Meat packing plant	LAB
*Pediococcus acidilactici*	ATCC 8081	Fermented milk	LAB
*Leuconostoc mesenteroides*	A5	Meat	LAB
*Lactobacillus bulgaricus*	ATCC11842	Yogurt	LAB
*Lactobacillus curvatus*	133L	Meat starter culture	LAB
*Lactobacillus sakei*	LB 808 (S206)	Unknown	LAB
*Carnobacterium piscicola*	M5L1	Vacuum package pork	LAB
*Carnobacterium divergens*	ATCC 35677	Vacuum package minced beef	LAB
*Aerococcus viridans*	ATCC 11563	Air sample	LAB
Generic *E. coli*	8_77	Meat packing plant	Spoilage
Generic *E. coli*	7_16	Meat packing plant	Spoilage
*Hafnia alvei*	S1	Vacuum-packaged meat	Spoilage
*Rahnella* sp.	S8	Vacuum-packaged meat	Spoilage
*Serratia* sp.	S10	Vacuum-packaged meat	Spoilage
*Sphingopyxis* sp.	03_68	Meat packing plant	Spoilage
*Comamonas koreensis*	25_64	Meat packing plant	Spoilage
*Raoultella terrigena*	ENT25_16	Meat packing plant	Spoilage
*Yersinia enterocolitica*	UN2814 602	Unknown	Spoilage
*Pseudomonas aeruginosa*	ATCC 7700	Well water	Spoilage
*Listeria monocytogenes*	GLM1	Meat-processing plant	Spoilage
*Listeria monocytogenes*	GLM3	Meat-processing plant	Spoilage

To simulate the nutrient profile within beef fabrication plants, LB-NS broth was supplemented with sterile beef purge (Pang and Yuk, 2018) that originated from vacuum-packed beef product (i.e., the eye of round). The vacuum package was opened, the beef purge was collected and distilled water was added at a ratio of 1:6. The aqueous solution was then passed through a 0.45 μm sterile filter (Midelet and Carpentier, 2002), protein content was determined using a Bradford kit (Thermo Scientific, Rockford, IL, United States) and the filtrate was stored at −20°C. The filtrate was mixed with LB-NS broth (10% v/v; mLB-NS) for use in biofilm formation assays.

### Biofilm-Forming Ability Determination

#### Crystal Violet Method Assessing Biofilm Formation

To assess biofilm formation, fresh cultures of each strain were diluted in mLB-NS to 10^6^ CFU/ml. Then, 200 μl of the 10^6^CFU/ml culture was transferred to designated wells in a 96-well microplate as described by Wang et al. (2018). Microplates were subsequently incubated at either 10 or 25°C for 6 days. At this point, microplates were washed three times with 300 μl of Butterfield’s Phosphate Buffer (BPB) per well using a microplate washer (405 LS, BioTek, Winooski, VT, United States). Washed plates were air-dried for 30 min in a biosafety level 2 cabinet (BSL2), and 200 μl of methanol was transferred to each well. After 15 min, the methanol was aspirated and 200 μl of 0.1% crystal violet (CV) was added to each well (Wang et al., 2016). After 15 min, the microplate was washed three times with 300 μl BPB per well, and the residual crystal violate in each well was solubilized in 200 μl of 85% ethanol (Wang et al., 2012). Biofilm-forming ability was determined indirectly by measuring residual chromophore using a microplate reader at 630 nm (BioTek ELx800; BioTek Instruments Inc., Winooski, VT, United States). Three repetitions were performed for each isolate (*n* = 16 × 3), with a total of 48 wells per isolate. Each isolate was categorized according to its biofilm-forming ability, with three microplate wells containing mLB-LS only serving as negative controls. The positive controls included *E. coli* O157:H7 R5O8, a known strong biofilm former (Adator et al., 2018).

To classify biofilm-forming ability, optical density cutoffs (ODc) were calculated as three standard deviations from the mean value of the control negative as described by Adator et al. (2018). Classifications included OD ≤ ODc = non-biofilm former; ODc < OD ≤ 2ODc = weak biofilm former; 2ODc < OD ≤ 4ODc = intermediate biofilm former; 4ODc < OD = strong biofilm former. The intermediate/strong biofilm formers identified at either 10 or 25°C were selected to form multispecies biofilms in subsequent experiments.

#### Shiga Toxin–Producing *Escherichia coli* Curli and Cellulose Expression

Shiga toxin–producing *E. coli* strains that possess curli fimbriae and produce cellulose are strong biofilm formers (Adator et al., 2018). To assess curli, fresh overnight cultures were plated onto Congo red agar (10 g/L casamino acids, 1 g/L yeast extract, and 20 g/L agar), supplemented with 20 μg/ml Coomassie brilliant blue dye (Sigma–Aldrich, St. Louis, MO, United States) and 40 μg/ml Congo red dye (Sigma–Aldrich), (CRI) (Adator et al., 2018). Cellulose production was assessed using fresh overnight cultures plated onto LB agar (Hardy Diagnostics CulGenex, Santa Maria, CA, United States) supplemented with 200 mg/L Calcofluor dye (Sigma–Aldrich) (Wang R. et al., 2013). Plates were incubated at 28°C for 72 h and cellulose production was assessed by measuring fluorescence at 366 nm (Wang R. et al., 2013). Duplicate samples were included in each experiment, with experiments replicated three times. Curli and cellulose production was defined as previously described (Gaylen et al., 2006; Wang R. et al., 2013) as follows:

(A) cellulose negative – no colony fluorescence at 366 nm; (B) cellulose positive – colony fluorescence at 366 nm; (C) curli negative – smooth and white colony; (D) curli positive – red, dry, and rough/brown, dry, and rough colonies (Supplementary Figure 1).

### Multispecies Biofilm Assays

#### Shiga Toxin–Producing *Escherichia coli* O103:H2 Multispecies Biofilm

**FIGURE 1:**
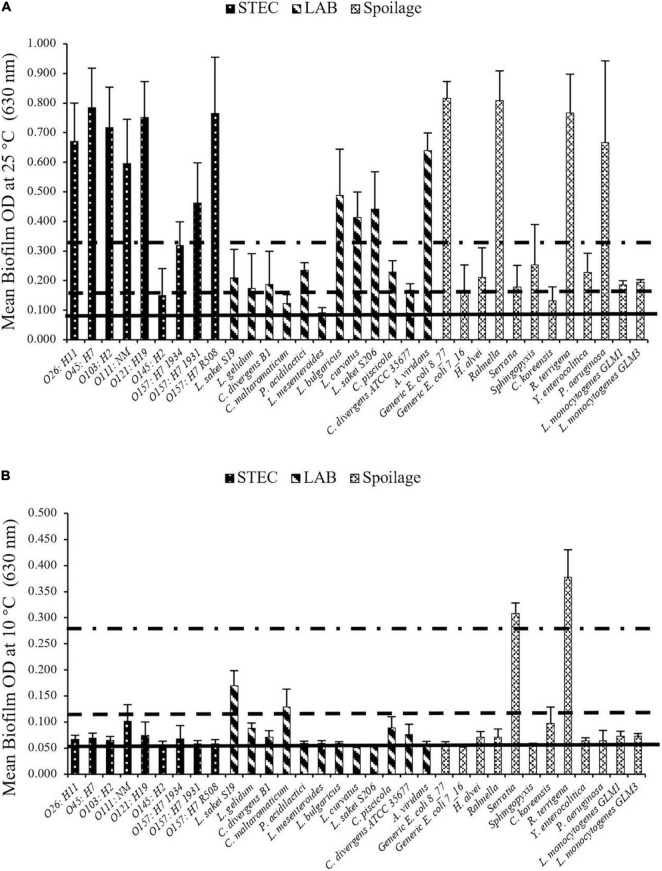
STEC biofilm formation on microplates after 6 days at **(A)** 25°C and **(B)** 10°C. Biofilms formed by each strain were determined in three replicate experiments. Horizontal lines going from bottom toward the top are non (OD < ODc), weak (ODc < OD < 2ODc), intermediate (2ODc < OD < 4ODc) and strong (4ODc < OD) biofilm formers. The biofilm forming ability differed (*P* < 0.001) with incubation temperature among strains.

Based on results obtained from the crystal violet assays, strong and intermediate biofilm formers were selected from STEC, LAB, and SP isolates for use in multispecies biofilm experiments (Figure 1). Four LAB (*Lactobacillus bulgaricus, Lactobacillus curvatus, Carnobacterium divergens* B1, and *Carnobacterium piscicola*) and one spoilage bacterium (*Pseudomonas aeruginosa*) were selected based on their ability to form strong/intermediate biofilms at 25°C (Figure 1A). Additionally, one LAB (*Lactobacillus sakei* S19) and three spoilage bacteria (*Serratia* sp., *Comamonas koreensis*, and *Raoultella terrigena*) were also selected based on their biofilm-forming ability at 10°C (Figure 1B). None of the STEC strains met the criteria as strong/intermediate biofilm formers at 10°C but several were strong biofilm formers at 25°C. Of these, O103:H2 (*stx*1 positive) was selected due to its high prevalence in fecal samples obtained from Canadian cattle just before slaughter (Stanford et al., 2016).

Lactic acid bacteria and SP bacterial mixed-biofilms were formed first and subsequently, O103:H2 was introduced into the mixed-species biofilm as described by Wang R. et al. (2013). Briefly, fresh cultures of each LAB and SP strain were diluted in mLB-NS to 10^6^ CFU/ml and then mixed according to the factorial arrangements in Table 2. Approximately 200 μl spoilage or LAB cultures (10^6^ CFU/ml) were aliquoted into microplate wells, with two sets of microplates for each experiment. Mature biofilms were allowed to form in the plates at 10 and 25°C over 6 days. After 6 days, the supernatant in each well was aspirated, and each well was washed with 200 μl BPB to remove free and loosely attached cells. At this point, 200 μl fresh O103:H2 culture (10^3^ CFU/ml) in mLB-NS was aliquoted into designated wells. Microplates were incubated for an additional 6 days, and thereafter washed three times with 300 μl BPB. One plate was used for enumeration and the other was used to assess the persistence O103:H2 in mixed biofilms. One column in the microplate was retained as a positive control with O103:H2 only, and a second column served as a negative control and received no inoculant. Each column (8 wells/column) was regarded as one observation, and the experiment was repeated three times in duplicate for each strain combination.

**TABLE 2:** Factorial design of strains combination.

	Lactic acid bacteria combination				
	***L. sakei* S19**	***C. divergens* B1**	***L. bulgaricus* ATCC11842**	***L. curvatus* 133L**	***C. piscicola* M5L1**
*L. sakei* S19					
*C. divergens* B1	+				
*L. bulgaricus* ATCC11842	+	+			
*L. curvatus* 133L	+	+	+		
*C. piscicola* M5L1	+	+	+	+	

	**Spoilage bacteria combination**				

	***Serratia* sp. S10**	***R. terrigena* ENT25_16**	***C. koreensis* 25_64**	***P. aeruginosa* ATCC7700**	
*Serratia* sp. S10					
*R. terrigena* ENT25_16	+				
*C. koreensis* 25_64	+	+			
*P. aeruginosa* ATCC7700	+	+	+		

#### Biofilm Shiga Toxin–Producing *Escherichia coli* Enumeration

Shiga toxin–producing *E. coli* enumeration was performed immediately after the microplate was washed. Buffered peptone water (200 μl; BPW, Hardy Diagnostics Inc.) was dispensed into each corresponding well and a sterile pipette tip was used to detach the biofilm by scraping the wall and bottom of each well (Wang R. et al., 2013). Subsequently, microplates were sonicated at 40 kHz (Branson 2800, Branson Ultrasonics Co., Danbury, CT, United States) for 1 min (Uhlich et al., 2006) and equal volumes of BPW from each well were pooled to generate 1 ml of culture for 10-fold dilution (Wang R. et al., 2013). O103:H2 was enumerated on MacConkey agar overlaid with TSA using the drop plate method (Herigstad et al., 2001). For the drop plate method, five drops (10 μl/drop) were dispensed on each plate, which was then incubated for 24 h at 37°C. Recovered colonies were confirmed as *E. coli* O103 *via* agglutination (SSI Diagnostica, Hillerød, Denmark) and PCR (Debroy et al., 2011).

#### Biofilm Shiga Toxin–Producing *Escherichia coli* Persistence and Survival

The second set of microplates containing multispecies biofilms was used to assess the survival of O103:H2 after desiccation. Microplates were maintained at 10 or 25°C for 1 month at ∼20–30% relative humidity (RH). Then, modified tryptone soy broth (200 μl; mTSB; Oxoid Ltd., Nepean, ON, Canada) was added to each well, and plates were incubated for 24 h at 37°C. A 3 μl aliquot of mTSB was removed from each well, spotted onto MacConkey agar, and verified as *E. coli* as described above.

### Multispecies Biofilm Formation on Food Contact Surfaces and Shiga Toxin–Producing *Escherichia coli* Transfer to Beef

#### Bacteria Strain and Culture Combination

**FIGURE 2:**
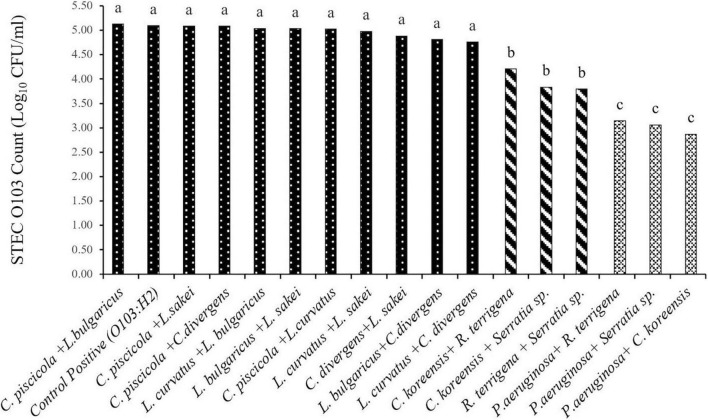
Least squares mean of STEC O103:H2 enumerated from 6-day old moist multispecies biofilms formed in microplates (SEM = 0.17). a,b,c: Least squares means with different superscript letter differ (*P* < 0.05).

Based on the O103:H2 cell numbers (Figure 2) and recovery rate (Table 3) from multispecies biofilms (*n* = 16), three species combinations were selected to form multispecies biofilm on food contact surfaces. These included T1: *C. piscicola* + *L. bulgaricus*; two SP combinations T2: *C. koreensis* + *R. terrigena* and T3: *P. aeruginosa* + *C. koreensis*. Biofilms were formed on thermoplastic polyurethane (TPU) and 304 stainless-steel (SS), common components of conveyor belts and food-processing surfaces, respectively.

**TABLE 3:** Recovery of STEC O103:H2 after 24 h of enrichment from multispecies dry biofilms stored for 30 days at 10 and 25°C.

Strain combination	Recover from 10°C, % (n/N)	Recover from 25°C, % (n/N)
*R. terrigena* + *Serratia* sp.	0.00 (0/6)	66.67 (4/6)
*C. koreensis* + *Serratia* sp.	50.00 (3/6)	83.33 (5/6)
*P. aeruginosa* + *Serratia* sp.	50.00 (3/6)	0.00 (0/6)
*C. koreensis* + *R. terrigena*	50.00 (3/6)	100.00 (6/6)
*P. aeruginosa* + *R. terrigena*	33.33 (2/6)	33.33 (2/6)
*P. aeruginosa* + *C. koreensis*	0.00 (0/6)	0.00 (0/6)
*C. divergens* B1 + *L. sakei* S19	0.00 (0/6)	100.00 (6/6)
*L. bulgaricus* + *L. sakei* S19	16.67 (1/6)	100.00 (6/6)
*L. curvatus* + *L. sakei* S19	0.00 (0/6)	100.00 (6/6)
*C. piscicola* + *L. sakei* S19	16.67 (1/6)	100.00 (6/6)
*L. bulgaricus* + *C. divergens* B1	16.67 (1/6)	100.00 (6/6)
*L. curvatus* + *C. divergens* B1	16.67 (1/6)	100.00 (6/6)
*C. piscicola* + *C. divergens* B1	16.67 (1/6)	100.00 (6/6)
*L. curvatus* + *L. bulgaricus*	33.33 (2/6)	100.00 (6/6)
*C. piscicola* + *L. bulgaricus*	33.33 (2/6)	100.00 (6/6)
*C. piscicola* + *L. curvatus*	0.00 (0/6)	100.00 (6/6)
Control positive (O103:H2)	50.00 (3/6)	33.33 (2/6)

#### Polyurethane and Stainless-Steel Coupons Preparation

Thermoplastic polyurethane coupons were prepared by sectioning a 2-ply white urethane smooth top surface food-grade conveyor belt (2E8U 0/02 White, NuTech Conveyor Components, Milton, CA, United States) into 2 cm × 2 cm pieces. Sanitized by soaking overnight in hydrogen peroxide (Accel PREVention, Diversey, Fort Mill, SC, United States). Coupons were then washed and soaked in sterile distilled water for 1 h.

Stainless steel 304 coupons (2 cm × 2 cm; Pegen Industries Inc., Stittsville, CA, United States) were washed in distilled water and placed in an ultrasonic water bath for 20 min at 60°C (Adator et al., 2018). Coupons were further sonicated in 15% phosphoric acid for 20 min at 60°C, an additional 20 min in distilled water, before dry-sterilization in an autoclave.

#### Dry and Wet Multispecies Biofilm Formation

Coupons were transferred to sterile Petri dishes (60 × 15 mm; VWR™, Radnor, PA, United States) and either the spoilage or LAB-mixed bacterial cultures (10^6^ CFU/ml) were added to each coupon (5 ml). Coupons were placed at either 10°C or 25°C, for 6 days to form mature biofilms. After 6 days, coupons were washed with BPB three times (10 ml/coupon) and placed in a new sterile Petri dish. Aliquots (5 ml) of O103:H2 culture (10^3^ CFU/ml) were then added to the preformed biofilms and incubated for an additional 6 days at each assigned temperature. Positive and negative controls were included as described above.

Coupons were stored under dry (20–50% RH) or moist conditions (60–90% RH), with moist biofilms being sprayed with sterile water (150 μl/coupon) daily. A subset of the TPU and SS coupons was used to determine the extent to which O103:H2 was transferred to beef. The second set of coupons was used for STEC enumeration from biofilms after 6, 30, and 60 days of storage.

#### Beef Samples Preparation to Test O103:H2 Transfer

Retail whole cut eye of round beef with the fat cap was purchased and kept at 4°C before use. A 5% lactic acid solution was used to wash the meat surface to reduce background flora and pieces were subsequently immersed in lactic acid for 1 min (Youssef et al., 2013). Beef pieces were allowed to drain and were cut into 3 cm × 3 cm pieces using an aseptic technique and stored at 4°C for up to 24 h.

#### Shiga Toxin–Producing *Escherichia coli* Transfer From Biofilms to Beef and Shiga Toxin–Producing *Escherichia coli* Biofilm Enumeration

For STEC transfer, beef pieces were placed on TPU or SS coupons, and a 50 g weight was placed on top of each piece to exert a pressure of 7.35 kPa (Flores et al., 2006). A piece of wax paper was placed between the weight and the meat to avoid direct contact with the weight during the 5 min contact time. Beef pieces were then removed from each coupon and placed in a Whirl-Pak bag (Nasco; Madison, WI, United States) along with 9 ml of BPW to obtain a 10-fold dilution and homogenized using a stomacher (Intersciences Inc., Markham, ON, Canada) for 1 min. For STEC enumeration, 10-fold dilutions were prepared and plated on TSA overlayed MacConkey agar (Wu, 2008). Plates were incubated for 24 h at 37°C and isolates were confirmed as described above.

To quantify STEC on TPU and SS, coupons were placed in whirl-Pak bags along with 9 ml of BPW to obtain a 10-fold dilution and sonicated for 1 min (Marouani-Gadri et al., 2009). For enumeration, the drop plate method was used as outlined above. Samples on MacConkey plates (undetectable levels) that did not produce colonies after 24 h at 37°C, were subject to enrichment in mTSB for 24 h at 37°C, before spread plating on MacConkey agar.

### Scanning Electron Microscopy

Scanning electron microscopy (SEM) was performed at the Manitoba Institute for Materials (MIM) to visualize dry biofilm formation on SS and TPU surfaces as described previously (Adator et al., 2018). The TPU and SS coupons were fixed (neutral buffered 10% formalin solution, Sigma–Aldrich) for 2 h and then washed with BPB for 30 min. The coupons were dried for 4 h at room temperature in a BSL2 cabinet, and the TPU surface was Gold–Palladium coated (Denton Vacuum Desk II, Moorestown, NJ, United States) in the high-vacuum mode on the following day. Biofilm structures were observed using a Quanta 650 FEG scanning electron microscope (FEI Co., Hillsboro, OR, United States) in the high-vacuum mode at 5 KV.

### Statistics Analysis

All experiments were performed three times. The Proc Mixed procedure of the Statistical Analysis System (Cary, NC, United States) was used to analyze the data with the least mean separation accomplished using the PDIFF option. For biofilm-forming ability and multispecies biofilm microplate assays, a factorial model was applied to analyze the main effects of bacterial strain, temperature, and their two-way interaction. For beef contaminated by O103:H2 on food contact surfaces, effects of species, contact surface, storage time, and humidity along with the appropriate interactions were tested. For all statistical analysis, significance was declared at *P* ≤ 0.05.

## Results

### Biofilm Formation Abilities Using the Crystal Violet Assay Method and Strain Selection

Isolates varied substantially in their biofilm-forming ability (Figure 1). A bacterial isolate by temperature interaction was identified (*P* < 0.001) as some isolates more readily formed biofilms at 25°C than others. For example, at 25°C many strains showed strong biofilm-forming abilities, which included all of the STEC strains except O145:H2 (weak) and O157:H7 1934 (intermediate), (Figure 1A). Within the LAB, *L. bulgaricus, L. curvatus, Lactobacillus sakei* S206, and *Aerococcus viridans* were all classified as strong biofilm formers at 25°C. Likewise, *P. aeruginosa, Rahnella* sp., *R. terrigena*, and *E. coli* (8_77) also formed strong biofilms at this temperature. In contrast, *Serratia* sp. and *R. terrigena* isolates were able to form strong biofilms at 10°C, while other isolates were intermediate or weak biofilm formers at this temperature (Figure 1B).

Evaluation of curli and cellulose indicated that strains O26: H11 (00-3941), O103: H2 (99-2076), O111: NM (CFS3), O121: H19 (03-2832), O157: H7 (R508), and *E. coli* (8_77) showed both curli- and cellulose-producing ability at 25°C (Table 4). Based on these data, *L. sakei* S19, *Serratia* sp., *C. koreensis, R. terrigena, L. bulgaricus, L. curvatus, C. divergens* B1, *C. piscicola, P. aeruginosa*, and O103:H2 were selected for further investigation in multi-species biofilms.

**TABLE 4:** Curli and cellulose production of the STEC and generic *E. coli* strains with different biofilm-forming abilities at 25°C.

Strain	Cellulose	Curli	Biofilm-forming ability
O26: H11 (00-3941)	+	+	Strong
O45: H7 (05-6545)	+	−	Strong
O103: H2 (99-2076)	+	+	Strong
O111: NM (CFS3)	+	+	Strong
O121: H19 (03-2832)	+	+	Strong
O145: H2 (75-83)	−	−	Weak
O157: H7 (1934)	−	−	Intermediate
O157: H7 (1931)	−	−	Strong
O157: H7 (R508)	+	+	Strong
Generic *E. coli* (8_77)	+	+	Strong
Generic *E. coli* (7_16)	−	−	Intermediate

### *In vitro* Multispecies Biofilms and Shiga Toxin–Producing *Escherichia coli* Interaction

#### Shiga Toxin–Producing *Escherichia coli* Enumeration From *in vitro* Multispecies Biofilms

Overall, O103:H2 numbers within the multispecies biofilm were affected (*P* < 0.001) by the strain combination (Figure 2). None of the tested LAB bacteria altered O103:H2 counts (*P* > 0.05) when compared with the positive control (5.10 log_10_ CFU/ml) and the numbers of O103:H2 recovered from LAB biofilms ranged from 4.76 to 5.13 log_10_ CFU/ml. When O103:H2 was exposed to SP biofilms, colonization by O103:H2 was reduced (*P* < 0.05), with the highest reduction (2.23 log_10_ CFU/ml) (*P* < 0.001) occurring with mixed *P. aeruginosa* and *C. koreensis* biofilms.

#### Shiga Toxin–Producing *Escherichia coli* Survival Within 30-Day Old Dry Multispecies Biofilms

After biofilms were kept dry for 30 days. *E. coli* O103:H2 was not recovered from the following biofilm combinations *P. aeruginosa* + *C. koreensis* and *P. aeruginosa* + *Serratia* sp. kept at 25°C (Table 3). Interestingly, the biofilm combination of *P. aeruginosa* + *R. terrigena* did not alter the recovery of O103:H2 (33%) as compared to O103:H2 single-species biofilms (33%). Regarding multispecies biofilms composed of LAB species, O103:H2 was recovered from all LAB biofilms (100%); interestingly, O103:H2 recovery from controls (O103:H2 single-species biofilms) was lower (33%) (Table 3). Similarly, O103:H2 was 100% recovered from mixed biofilms containing *C. koreensis* + *R. terrigena*.

In contrast, at 10°C, O103:H2 recovery from all mixed-species biofilms was much lower (0–50%) than at 25°C (Table 3). Interestingly, when looking at controls, *E. coli* O103 was better able to survive within biofilms formed and kept at 10°C than from within those formed and kept at 25°C (50% survival vs. 33.33% survival). However, some multispecies biofilms were able to reduce O103:H2 recovery to 0%, including *R. terrigena* + *Serratia sp., P. aeruginosa* + *C. koreensis*, and *C. divergens* B1 + *L. sakei* S19. While other combinations showed no effect on survival (Table 3).

### Multispecies Biofilm Formation on Food Contact Surfaces and Shiga Toxin–Producing *Escherichia coli* Transfer to Fresh Beef

**FIGURE 3:**
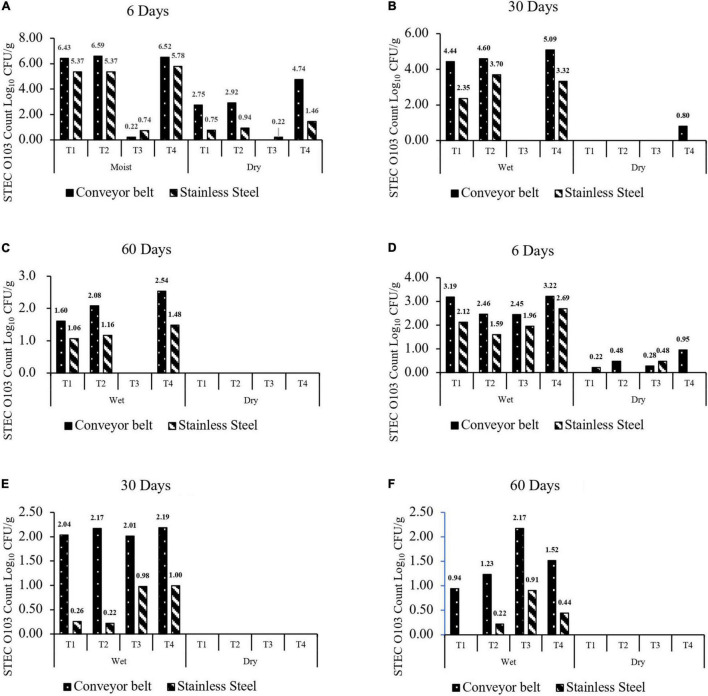
**(A–C)** Number of O103:H2 cells (CFU/g) transferred to beef from moist or dry multispecies biofilms formed at 25°C for 6, 30, and 60 days. **(D–F)** Number of O103:H2 cells (CFU/g) transferred to beef from moist or dry multispecies biofilms formed at 10°C for 6, 30, and 60 days. The four-strain combination were (T1) C. *piscicola* + *L. bulgaricus*; (T2) C. *koreensis* + *R. terrigena*; (T3) *P aeruginosa* + C. *koreensis*; and (T4) STEC O103:H2 Control. The limit of detection was 1 log_10_ CFU/g.

At 25°C, spoilage of *P. aeruginosa* + *C. koreensis* (T3) biofilms reduced (*P* < 0.001) the transfer of O103:H2 to beef by 2.54 log_10_ CFU/g (Figures 3A–C). Mixed species *C. piscicola* + *L. bulgaricus* (T1) and *C. koreensis* + *R. terrigena* (T2) biofilms did not alter (*P* > 0.05) the transfer of O103:H2 cells to beef. Overall transfer of O103:H2 to beef from biofilms formed on TPU (2.14 log_10_ CFU/g) was greater (*P* < 0.001) than that from SS (1.40 log_10_ CFU/g). Transfer of O103:H2 to beef decreased (*P* < 0.001) with biofilm aging, from 3.17 log on Day 6 to 1.52 log_10_ CFU/g on Day 30 and 0.62 log_10_ CFU/g on Day 60. Reductions in the transfer of O103:H2 to beef were highest for 6 days T3-mixed biofilms grown on TPU. Overall moist biofilms transferred more O103:H2 (*P* < 0.001) to beef (2.93 log_10_ CFU/g) than dry biofilms (0.61 log_10_ CFU/g) regardless of the surface type.

At 10°C, none (*P* > 0.05) of the multispecies biofilms reduced or enhanced the transfer of O103:H2 to beef as compared to the control positive (Figures 3D–F). Transfer of O103:H2 to beef was also higher (*P* < 0.001) from TPU (1.14 log_10_ CFU/g) than from SS (0.55 log_10_ CFU/g). Results also showed that moist biofilms were more (*P* < 0.001) likely to contaminate beef (1.58 log_10_ CFU/g) than dry biofilms (0.10 log_10_ CFU/g). At 10°C, contamination of beef with O103:H2 decreased (*P* < 0.001) as biofilms aged, from 1.38 log_10_ CFU/g after 6 days to 0.47 log_10_ CFU/g after 60 days.

### O103:H2 Recovery From Dry Biofilms

At 25°C, no O103:H2 was recovered (0%) from 60 days dry biofilms on SS, even after enrichment (Table 5). On TPU, O103:H2 was recovered from approximately 33% of dry 60 days multispecies of *C. koreensis* + *R. terrigena* (T2) and control positive (T4) biofilms. However, O103:H2 was not recovered from the following multispecies biofilms combinations *C. piscicola* + *L. bulgaricus* (T1) and *P. aeruginosa* + *C. koreensis* (T3).

**TABLE 5:** Recovery of STEC O103:H2, with and without enrichment from dry multispecies biofilms stored at 25 or 10°C for 60 days.

Surface	Strain combination	Recover without enrichment, % (n/N)	Recover with enrichment, % (n/N)	Total recover, % (n/N)
25°C				
TPU	T1: *C. piscicola* + *L. bulgaricus*	0.00 (0/9)	0.00 (0/9)	0.00 (0/9)
	T2: *C. koreensis* + *R. terrigena*	0.00 (0/9)	33.33 (3/9)	**33.33 (3/9)**
	T3: *P. aeruginosa* + *C. koreensis*	0.00 (0/9)	0.00 (0/9)	0.00 (0/9)
	T4: Control positive (O103:H2)	0.00 (0/9)	33.33 (3/9)	**33.33 (3/9)**
SS	T1: *C. piscicola* + *L. bulgaricus*	0.00 (0/9)	0.00 (0/9)	0.00 (0/9)
	T2: *C. koreensis* + *R. terrigena*	0.00 (0/9)	0.00 (0/9)	0.00 (0/9)
	T3: *P. aeruginosa* + *C. koreensis*	0.00 (0/9)	0.00 (0/9)	0.00 (0/9)
	T4: Control positive (O103:H2)	0.00 (0/9)	0.00 (0/9)	0.00 (0/9)
10°C				
TPU	T1: *C. piscicola* + *L. bulgaricus*	0.00 (0/9)	33.33 (3/9)	33.33 (3/9)
	T2: *C. koreensis* + *R. terrigena*	11.11 (1/9)	87.50 (7/8)	**88.89 (8/9)**
	T3: *P. aeruginosa* + *C. koreensis*	0.00 (0/9)	22.22 (2/9)	22.22 (2/9)
	T4: Control positive (O103:H2)	0.00 (0/9)	44.44 (4/9)	44.44 (4/9)
SS	T1: *C. piscicola* + *L. bulgaricus*	0.00 (0/9)	11.11 (1/9)	11.11 (1/9)
	T2: *C. koreensis* + *R. terrigena*	0.00 (0/9)	33.33 (3/9)	**33.33 (3/9)**
	T3: *P. aeruginosa* + *C. koreensis*	0.00 (0/9)	0.00 (0/9)	0.00 (0/9)
	T4: Control positive (O103:H2)	0.00 (0/9)	11.11 (1/9)	11.11 (1/9)

*The value which was highlighted in font bold was applied to indicate a higher or equal O103:H2 recovery rate when comparing with control positive (T4).*

At 10°C after enrichment, O103:H2 was most often recovered from mixed biofilms of T2 formed on TPU/SS and stored for 60 days (Table 5). Recoveries of O103:H2 from dry biofilms on TPU ranked as T2 (89%) > T4 (44%) > T1 (33%) > T3 (22%). On SS surfaces, the highest O103:H2 recovery rate from dry biofilm was as follows T2 (33%) > T4 (11%) = T1 (11%) > T3 (0%).

### Scanning Electron Microscopy

**FIGURE 4:**
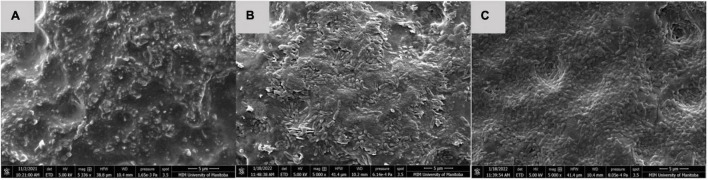
SEM of **(A)** TPU coupon used as control negative, no bacteria observed. **(B)** T2: *C*. *koreensis* + *R. terrigena*; and **(C)** T4: STEC O103:H2 Control positive 25°C dry biofilm at day 60 on a TPU surface. In panels **(B,C)** a well-developed multilayered biofilm is displayed; the rod-shaped bacteria are dominant in biofilm and covered within the extensive EPS matrix.

**FIGURE 5:**
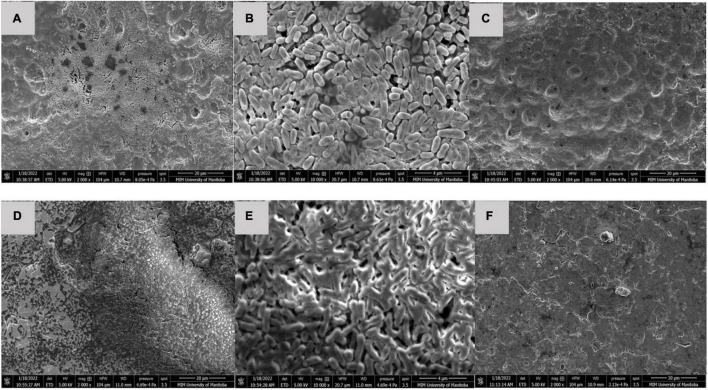
SEM of **(A,B)** T2: *C*. *koreensis* + *R. terrigena;* and **(C)** T4: STEC OI03:H2 Control positive 10°C dry biofilm at day 60 on a TPU surface. **(D,E)** T2: *C*. *koreensis* + *R. terrigena*; and **(F)** T4: STEC O103:H2 Control positive 10°C dry biofilm at day 60 on a SS surface. In panels **(A,B,D,E)** 60 day dry biofilms are shown at on TPU and SS surface, the biofilm is dominated by rod shaped bacteria with the EPS matrix; a well-developed multilayered biofilm displayed, which covered the TPU and SS surface. In panels **(C,F)** no individual bacteria and EPS displayed on the surface of TPU and SS coupons.

After 60 days storage, dry multispecies biofilms composed of *C. koreensis* + *R. terrigena* (T2) were observed as a multilayer structure with rod-shaped bacteria covered by extensive EPS at both 25°C (Figure 4) and 10°C (Figure 5). Interestingly, control positive (T4) dry biofilm was displayed differently after 60 days storage at 25°C (Figure 4) and 10°C (Figure 5). At 25°C, a well-developed multilayer T4 biofilm extensively covered the whole TPU surface. However, no individual bacteria and EPS were observed when the T4 biofilm was stored at 10°C for 60 days.

## Discussion

### Biofilm Forming Ability

In this study, strains isolated from beef-processing facilities (e.g., *L. sakei* S19, *C. maltaromaticum* and *R. terrigena*) and meat products (*Serratia* sp.) were found to be stronger biofilm formers at 10°C than those (e.g., *L. bulgaricus* and *L. curvatus*) isolated from the fermented product. This is showing that isolates from beef production facilities are likely adapted to growth and form biofilms at low temperatures (Visvalingam et al., 2019a). In Canada, beef fabrication facilities operate at temperatures below 10°C to limit the growth of enteric pathogens and spoilage bacteria (Visvalingam et al., 2017; Yang et al., 2017a,b). However, temperatures at the beef fabrication facility vary and can reach up to 15°C during non-operational hours (Visvalingam et al., 2017). In addition, the equipment used during beef fabrication and other interventions can generate higher temperatures, such as frictional heat produced by conveyor belts or the transfer of body heat to gloves, and the application of high-pressure hot water (40–50°C) during sanitation. Thus temperature variation and microenvironments created in the beef-processing facility attributed to different factors could promote the formation of biofilms within the beef-processing environment (Yang et al., 2015, 2017a; Visvalingam et al., 2016).

### Shiga Toxin–Producing *Escherichia coli* Curli and Cellulose Production Determination

Curli fimbriae and the production of cellulose have been reported to play a significant role in STEC biofilm formation and persistence (Gualdi et al., 2008; Iibuchi et al., 2010; Adator et al., 2018). Curli play a significant role in mediating surface and cell-to-cell contact in *E. coli* and *Salmonella* biofilms (Barnhart and Chapman, 2006). Uhlich et al. (2014) demonstrated that curli and cellulose formation are influenced by temperature and media composition (Uhlich et al., 2014). Results demonstrate STEC strain O145:H2 (75–83), and O157:H7 (1934) showed curli-producing ability at 37°C instead of at 25°C, while only STEC O157:H7 (R508) and *E. coli* (8_77) kept cellulose-producing capacity at 37°C (Supplementary Table 1). Except for the strong biofilm former O157:H7 (1931), STEC lacked curli or did not produce cellulose and were only able to form weak or intermediate biofilms at 25°C (Table 4). Wang et al. (2012), also reported that the occasional strain of STEC that lacked curli could still produce strong biofilms at room temperature (Wang et al., 2012). Stanford et al. (2021) also found that some STEC strains that produced curli did not form strong biofilms. This indicates that traits other than just curli and cellulose production are likely to mediate biofilm formation in some STEC strains (Wang et al., 2012; Visvalingam et al., 2017). For instance, some research has shown that biofilm formation accompanied by massive EPS production is highly reliant on *E. coli* metabolic energy support (Landini, 2009). However, energy metabolisms among the top six STEC serogroups have been reported to be highly diverse, which might be attributed to the genetic diversity among serogroups (Chen et al., 2020).

### Contamination of Food Contact Surfaces by Multispecies Biofilms

Some studies have shown that *E. coli* found on the surface of fabrication equipment can survive sanitation procedures and contaminate meat (Yang et al., 2015; Visvalingam et al., 2016). STEC attached to food contact surfaces may also interact with pre-established multispecies biofilm (Wang, 2019). In the present study, well-structured multilayer multispecies biofilms were developed (Figures 4, 5) and O103:H2 within these biofilms was able to transfer to beef (Figure 3), Furthermore, Visvalingam et al. (2019a) demonstrated that STEC O157:H7 readily integrated (3.8 log_10_ CFU/cm^2^) into multispecies biofilms containing 41 different bacterial strain isolated from beef-packing plant (Visvalingam et al., 2019a). In the same study, the relative abundance of participating strains (*n* = 42) did vary; as some strains such as *Carnobacterium* sp. accounted for (10%) of the biofilm membership, whereas O157:H7 accounted for only 0.04% of the community (Visvalingam et al., 2019a).

Contamination of beef by O103:H2 was substantially reduced after interaction with *P. aeruginosa* + *C. koreensis* (T3) biofilms. Both *Pseudomonas* sp. (Pang et al., 2017; Pang and Yuk, 2018) and *Comamonas* sp. (Carpentier and Chassaing, 2004) have been shown to form robust biofilms on stainless steel surfaces. In the present study, pre-existing biofilms of *P. aeruginosa* + *C. koreensi*s on TPU and SS may have inhibited O103:H2 integration into biofilms. Wang et al. (2015) investigated mixed biofilms of STEC serotypes O157:H7 and O111:H8 and found that the STEC serotype that was inoculated onto the surface first, exhibited the dominant membership within mature biofilms (Wang et al., 2015). Most biofilm studies have inoculated similar numbers of different bacterial species onto food contact surfaces (Wang R. et al., 2013; Liu et al., 2014; Pang et al., 2017). Within beef fabrication facilities, probably multispecies biofilms are already established on contact surfaces (Wang, 2019). These mature biofilms may preclude the integration of STEC due to a lack of adhesion sites or available nutrients (Pang and Yuk, 2018). Hence, developing biofilms composed of pre-selected innocuous bacterial species may inhibit STEC biofilm formation and have merit as a biocontrol strategy (Alegre et al., 2013). On the other hand, *C. piscicola* + *L. bulgaricus* (T1) and *C. koreensis* + *R. terrigena*. (T2) biofilms did not affect the extent that O103:H2 contaminated beef. The impact of mixed-species biofilms on the contamination of meat is likely species dependent and possibly strain dependent (Wang, 2019). For example, *Pseudomonas* sp. have been shown to inhibit the formation of *E. coli* O157:H7 biofilms on SS (Kim et al., 2018) and *Salmonella* biofilms on TPU (Wang H.-H. et al., 2013). *Pseudomonas* sp. are known to produce antimicrobials such as pyocyanin, pyoluteorin, and siderophores, which may inhibit the integration of foreign bacteria into biofilms (Hernández-León et al., 2015; Collazo et al., 2017; Kim et al., 2018). Moreover, *P. aeruginosa* may also produce acyl homoserine-lactones (AHL) (Lee et al., 2007), which have been shown to inhibit *E. coli* biofilm formation by altering gene expression (Van Houdt et al., 2006).

### Interactions of Lactic Acid Bacteria Multispecies Biofilms With O103:H2

Certain groups of LAB such as *Lactobacillus* sp. are commonly used as probiotics, as many of these isolates produce bacteriocins and organic acids (e.g., lactic acid) (Schrezenmeir and De Vrese, 2001; Imani Fooladi et al., 2014). Previous research indicates that some *Lactobacillus* spp. can reduce the shedding of *E. coli* O157:H7 in cattle feces (Brashears et al., 2003; Younts-Dahl et al., 2004). If added to ground beef at 5°C, they can also reduce the prevalence of *E. coli* O157:H7 and *Salmonella* (Smith et al., 2005). *Carnobacterium* sp. are frequently isolated from beef fabrication facilities, and can persist on non–food-contact surfaces after sanitization (Wang et al., 2018). Interestingly, multiple studies indicate that *Carnobacterium* sp. can inhibit the growth of *Listeria monocytogenes* on meat by producing bacteriocins, but this species can also cause food spoilage (Leisner et al., 2007). In the present study, no synergistic or antagonistic interactions on beef contamination were observed between O103:H2 and *C. piscicola* + *L. bulgaricus* biofilms. Furthermore, extracts of *C. piscicola* and *L. bulgaricus* did not exhibit activity against O103:H2 in clearing zone assays (data not shown). Most LAB bacteriocins target a narrow range of bacteria and primarily target Gram-positive bacteria such as *Listeria* (Jones et al., 2008). Jones et al. (2008) tested 75 meat-related LAB strains and none of them showed antimicrobial activity against *E. coli* O157:H7 (Jones et al., 2008). Others have shown that *Lactobacillus* sp. and *Lactococcus* sp. bacteriocin activity against *E. coli* is strain dependent (Gómez et al., 2016). *Lactobacillus* sp. can also produce lactic, acetic, and propionic acids, which can inhibit the growth of pathogenic bacteria (Abedi et al., 2013; Jalilsood et al., 2015). However, after 6 days the alkaline extract (pH > 8) was produced by *C. piscicola* + *L. bulgaricus* (T1) biofilms, suggesting that the production of ammonia from amino acid metabolism may have neutralized any antimicrobial activity of organic acids.

### The Beef Contamination by O103:H2 Varies on Different Food Contact Surfaces

Stainless steel and thermoplastics are two of the most common food contact surfaces used in the food industry (Chia et al., 2009; Sofos and Geornaras, 2010). In this research, the transfer of O103:H2 to beef from biofilms on TPU was greater than that from SS (Figure 3). In previous studies, *E. coli* O157:H7 and *L. monocytogenes* were able to form stronger monoculture biofilms (higher bacteria count) on polyurethane plastic than on stainless steel (SS-304) (Midelet and Carpentier, 2002; Graziella et al., 2006; Sofos and Geornaras, 2010). The stronger biofilms on polyurethane surfaces may be related to its greater hydrophobicity than stainless steel (Donlan, 2002). It has been hypothesized that as bacteria cells irreversibly attach to solid surfaces, hydrophobic surfaces may have less electrostatic repulsive forces (Loosecht et al., 1987; Sinde and Carballo, 2000; Donlan, 2002). Other bacteria such as *Salmonella* and *Listeria* have also been found to more readily attach and form biofilms on surfaces that are more hydrophobic (Sinde and Carballo, 2000; Donlan, 2002). Apart from beef contamination, the different food contact surfaces also affected O103:H2 recovery from 60 days dry biofilms (Table 5), with O103:H2 being more readily recovered from multispecies on TPU than SS at both 10 and 25°C. A similar result has been reported by Adator et al. (2018), as recovery rates from STECs biofilms were higher on polystyrene than SS (Adator et al., 2018). Previous studies have demonstrated that conveyor belts are often linked to the contamination of beef trimmings and cuts with *E. coli* in beef fabrication facilities (Youssef et al., 2013; Yang et al., 2015; Visvalingam et al., 2016). Regarding the efficiency of routine commercial sanitation processes in beef-processing facilities, published work shows that the sanitation process cannot completely remove *E. coli* from the conveyor belt (Yang et al., 2015, 2017b; Visvalingam et al., 2016). This limited *E. coli* removal has been attributed to meat residues, which can reduce the efficacy of sanitizers and the impact of desiccation on the viability of *E. coli* (Yang et al., 2015, 2017b; Visvalingam et al., 2016). Our study did find that O103:H2 within 60 days dry biofilms did not transfer to beef (Figure 3). In contrast, O103:H2 bacteria within moist biofilm readily transferred to beef even after 60 days of storage at 10 or 25°C (Figure 3). As indicated by Gill and Landers (2004), others have shown that desiccation can reduce the transfer of *E. coli* from fabrication equipment to beef (Gill and Landers, 2004; Youssef et al., 2013). However, humid conditions are prevalent in the beef industry due to condensation originating from the routine use of hot water during the sanitation processes (Møretrø et al., 2010), which could re-hydrate dry biofilms allowing bacteria within the biofilm to thrive, persist, and spread. Typically, the relative humidity within beef-processing plants is high, varying from 40 to 97% during the day with peak humidity reached during sanitation (Møretrø et al., 2010). Meat residues on the conveyer belts, combined with the high relative humidity are factors that undoubtedly contribute to the formation of robust surface biofilms (Yang et al., 2015, 2017b; Visvalingam et al., 2016) or maintenance of dry old biofilms. Results obtained in this research showed that prolonged storage time was associated with a decrease in transfer to O103:H2 from biofilms to beef at both 10 and 25°C. During prolonged storage, nutrients within biofilms may become limiting and a buildup of waste products may also reduce cell viability within biofilms (Rendueles and Ghigo, 2015). However, dry biofilms can pose a cross-contamination risk if those dry biofilms come in contact with meat juices and water since STEC can still be viable within the biofilm.

### The O103:H2 Persistence in Dry Multispecies Biofilm During Long Periods of Storage

Although O103:H2 associated with 60 days dry biofilms did not transfer to beef, viable O103:H2 could still be recovered from dry biofilm after enrichment (Table 5). Others have also found that dormant STEC cells can be recovered from dry biofilms *via* enrichment, which mimics the conditions at the meat plants where dry biofilms could rehydrate with water and beef juices allowing bacteria to recover (Adator et al., 2018). After enrichment, O103:H2 recovery from *C. koreensis* + *R. terrigena* (T2)-mixed biofilm at 10°C was always higher than from positive control (T4) 60 days dry biofilms (Table 3). Furthermore, SEM images indicated that 10°C. *koreensis* + *R. terrigena* (T2) 60 days dry biofilm displayed well-structured multilayered biofilm on TPU and SS surfaces (Figure 5). Instead, 10°C positive control (T4) 60 days dry biofilm was non-observed on both surfaces (Figure 5). This is showing that spoilage bacteria naturally occurring in beef-processing environments, that could survive the sanitation process and are adapted to lower temperatures, could shelter STEC and allow its persistence. Other researchers have made similar observations for *Pseudomonas*–*Salmonella* mixed biofilms, where the presence of *P. aeruginosa* enhanced *Salmonella typhimurium* and *Salmonella enteritidis* resistance to sanitizers (Pang et al., 2017). *P. aeruginosa* has been shown to produce more EPS (e.g., glycoconjugates) in mixed-species biofilms, thicker EPS hinders sanitizer penetration and thus protects *Salmonella* against sanitizers (Pang et al., 2017; Pang and Yuk, 2018). During biofilm development, EPS are secreted by the bacterial community, complex matrixes are formed which in turn embed bacterial cells (Flemming and Wingender, 2010) and protect them from desiccation while trapping nutrients (Kumar and Anand, 1998; Stewart and Franklin, 2008). Therefore, it is possible that *C. koreensis* + *R. terrigena* (T2) produced a more complex EPS matrix at 10°C (Figure 5) that enhanced the ability of O103:H2 to persist in desiccated multispecies biofilms. In beef-processing facilities, dry biofilms on beef fabrication equipment that come in contact with beef purge or water may result in conditions that promote cell viability similar to enrichment (Skandamis et al., 2009; Adator et al., 2018). If this is the case, even old dry biofilms on beef fabrication equipment could continuously pose a risk of beef contamination (Skandamis et al., 2009; Yang et al., 2017a; Adator et al., 2018).

To limit enteric pathogen growth on food contact surfaces and the product low environmental temperatures (5–15°C) is maintained in the beef industry (Ma et al., 2019, 2020). Our research shows that a temperature of 10°C reduced O103:H2 biofilm formation and thus cell transfer to beef when compared with biofilms formed and kept at 25°C. However, a higher O103:H2 recovery rate was observed in 60 days dry biofilms formed and maintained at 10°C instead of 25°C, the highest O103:H2 recovery was obtained from *C. koreensis* + *R. terrigena* (T2)-mixed biofilm at 10°C. Interestingly, both strains, *C. koreensis* and *R. terrigena* can form biofilms at 10°C. This finding suggests that despite reducing STEC biofilm formation in beef fabrication facilities through temperature control, STEC can still pose a cross-contamination risk to beef, especially under the assistance of psychotrophic spoilage bacteria in the beef-processing environment.

Scanning electron microscope imaging was used to verify the biofilm formation on the food contact surface, important to mention that one of the disadvantages of using SEM to research biofilms is that biofilm structure could be altered due to the fixation procedures, mainly when studying wet biofilms, however, in this research biofilm structure was not part of the objectives. The present study focused on STEC, spoilage, and LAB interactions, mainly to assess STEC cross-contamination potential. Multispecies biofilm structure or composition was not part of our research objectives. Further research is needed to investigate the dynamic spatial distribution of STEC within the multispecies biofilm, for which different microscopy techniques are needed, such as confocal laser scanning microscopy (CLSM) (Chen et al., 2015). In addition, multiple latest studies indicate that the metabolic diversity among STEC serogroups might lead to varied stress responses (Chen et al., 2020; Wang et al., 2022; Zhao et al., 2022), suggesting the interaction in multispecies biofilm may be varied according to different STEC serogroups. Hence, further studies could attempt to investigate the general interaction between SP or LAB multispecies biofilm with STEC (e.g., top 7 STEC) and also could look into STEC genetic makeup. Such work could facilitate the development of biofilm management strategies for STEC in beef-processing environments.

## Conclusion

Bacteria commonly found in the food industry played a significant role in STEC persistence and survival. The biofilm mixture *P. aeruginosa* + *C. koreensis* was the most antagonistic toward O103:H2 at 25°C, and *C. koreensis* + *R. terrigena* dry biofilms showed the highest recovery of O103:H2 at 10°C. Moreover, LAB biofilm did not reduce the extent to which O103:H2 was transferred from biofilms to beef, which may indicate that the interaction between O103:H2 and pre-developed biofilm is strain-dependent. Conditions for multispecies biofilm formation, including humidity, adherent surface, and storage time are variables, that played significant roles in beef contamination by O103:H2. Beef contamination with O103:H2 was more severe when it contacted fresh moist biofilms on TPU. Thus, further improvements for cleaning conveyor belts should be explored since scientific data are indicating that conveyor belt material allows biofilm formation and persistence. Perhaps developing different materials less prone to bacterial attachment and colonization could be explored. Furthermore, O103:H2 biofilm formation reduced at low temperatures; however, a higher STEC recovery from 10°C dry biofilms were observed. Results suggest that STEC persistence may not only depend on biofilm-forming ability but also be related to the bacteria community in the beef-processing environment. Findings in the present study confirm that development of SP or LAB multispecies biofilms with O103:H2 can either increase or diminish the likelihood of beef contamination.

## Data Availability Statement

The original contributions presented in the study are included in the article/Supplementary Material, further inquiries can be directed to the corresponding author.

## Author Contributions

CN-B and TM developed this project. CN-B supervised the students and lab work. YN designed the study and performed the experiment. AR-G analyzed the data. YN drafted the manuscript with the helps from KS, CN, XY, TM, and CN-B. All authors listed have made a significant and direct contribution to this project and approved it for publication.

## Conflict of Interest

The authors declare that the research was conducted in the absence of any commercial or financial relationships that could be construed as a potential conflict of interest.

## Publisher’s Note

All claims expressed in this article are solely those of the authors and do not necessarily represent those of their affiliated organizations, or those of the publisher, the editors and the reviewers. Any product that may be evaluated in this article, or claim that may be made by its manufacturer, is not guaranteed or endorsed by the publisher.
