# Supplementary figures and images for: Formation and Transfer of Multi-Species Biofilms Containing E. coli O103:H2 on Food Contact Surfaces to Beef
Source: Front Microbiol. 2022 May 30;13:863778. doi: 10.3389/fmicb.2022.863778 (PMC9196126; doi:10.3389/fmicb.2022.863778)

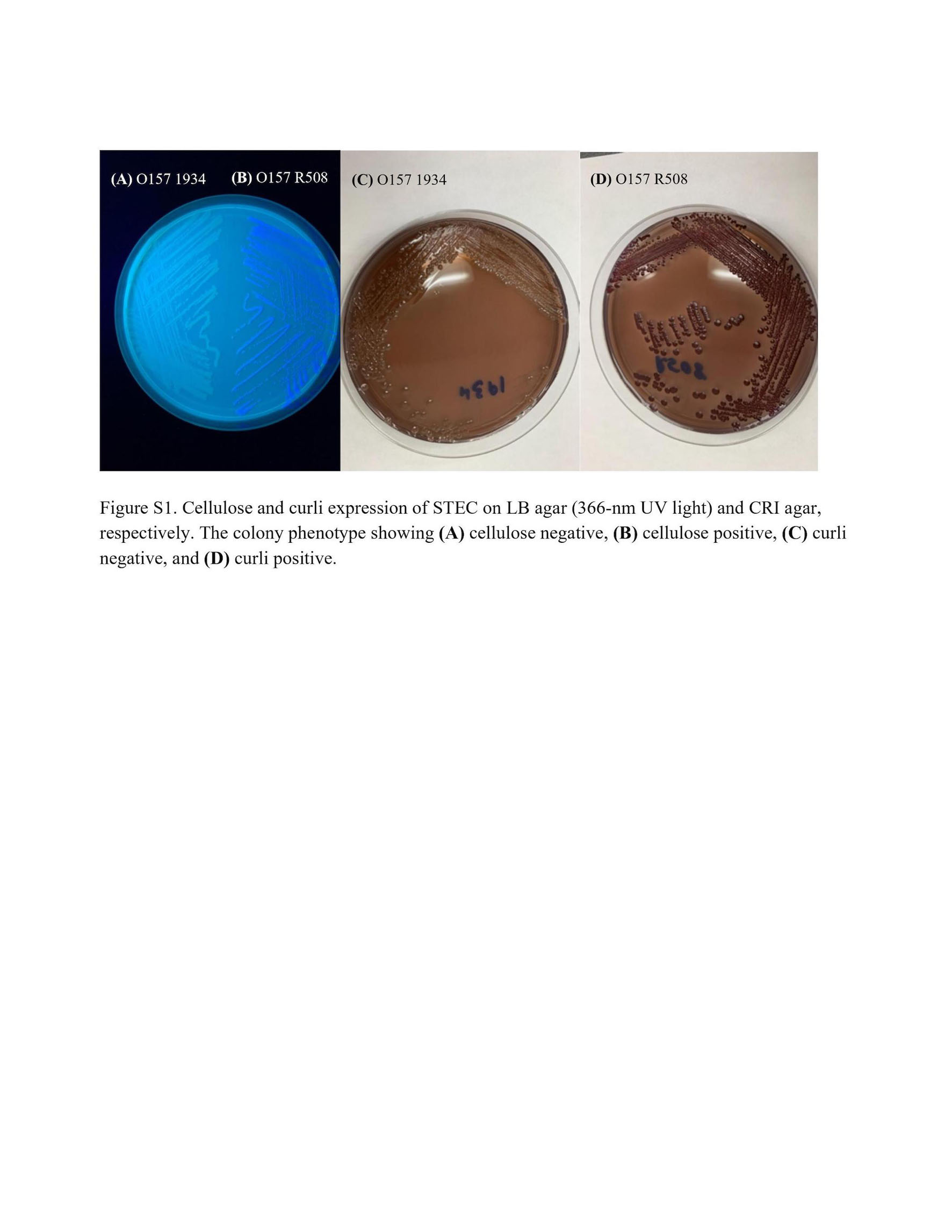

Supplement: Supplementary file 1 [file Image_1.JPEG]
