# Supplementary material for: Formation and Transfer of Multi-Species Biofilms Containing E. coli O103:H2 on Food Contact Surfaces to Beef
Source: Front Microbiol. 2022 May 30;13:863778. doi: 10.3389/fmicb.2022.863778 (PMC9196126; doi:10.3389/fmicb.2022.863778)
Supplement: Supplementary file 2 [file Table_1.DOCX]

Supplementary Material

Table S1. Curli and cellulose production of the STEC and generic *E. coli* strains at 37 °C.

| Strain | Cellulose | Curli |
| --- | --- | --- |
| O26: H11 (00-3941) | - | + |
| O45: H7 (05-6545) | - | - |
| O103: H2 (99-2076) | - | + |
| O111: NM (CFS3) | - | + |
| O121: H19 (03-2832) | - | + |
| O145: H2 (75-83) | - | + |
| O157: H7 (1934) | - | + |
| O157: H7 (1931) | - | - |
| O157: H7 (R508) | + | + |
| Generic *E. coli* (8_77) | + | + |
| Generic *E. coli* (7_16) | - | - |
